# Criticism of the National Association of Dental Faculties

**Published:** 1904-03-15

**Authors:** L. Greenbaum


### CRITICISM OF THE NATIONAL ASSOCIATION OF DENTAL FACULTIES.

In the President's Address, delivered by Dr. J. D. Patterson, before the Institute of Dental Pedagogics, at Buffalo, N. Y., in December, 1903 (published in *Items of Interest*, February, 1904), the doctor makes rather a strong arraignment of the National Association of Dental Faculties, which, he says, "*is a legislative body which too often does not legislate; an administrative body that does not administer*, and whose requirement of obedience to rules is elastic, varied to suit the individual case."

The doctor further says: "The desired work of improving the standard of the graduate cannot be left to that association, for it has not been faithful to its trust. I speak with all calmness and certitude, and the record will substantiate me in stating the claim that since the Faculties Association in 1897 in an illegal manner abandoned the advanced standard which it had adopted in 1896, and which advanced standards in their elaboration would unquestionably have brought a rapid advance in the professional standard in dentistry, that association cannot be credited with any advance toward making our profession dignified and professional. The average intelligence of the student in late years has not improved, and a same dead level of indifference to everything but a diploma and the money it will enable them to grasp exhibits itself in the great majority of those we are called upon to instruct."

We cannot agree with the doctor in reference to the cause of existing conditions; nor can we join him in his pessimistic views concerning the results. It is our opinion that the National Association of Dental Faculties is a legislative body which too often legislates too much and not too little. If any fault may be found with its proceedings it would be with the excess of legislation—(which tends to confusion)—rather than its deficiency. And, concerning the lax administration of rules, which the doctor deplores, we feel that the association as a body can hardly be blamed for that, because it has no means of enforcing its rules beyond the sense of moral obligation felt by its members to live up to the rules formulated. We are impressed with this fact very strongly, because we have had a good opportunity of observing the working of the rules; particularly those concerning entrance requirements in our own State. The schools in Pennsylvania live up to the rule concerning entrance requirements both in letter and spirit, and have placed the matter of examining entrance credentials fully and completely in the hands of the State authorities. This insures rigid and equal enforcement by all schools, and in consequence of this we have observed in our students (we refer to the schools in Pennsylvania) a higher grade of intelligence, and a demand for instruction in keeping therewith. On the other hand, with those who come to us from neighboring States, where men are admitted to the earlier courses without a strict surveillance of entrance requirements and afterward unloaded on other colleges, that such students labor under great disadvantages, that they suffer in comparison with those who have been untrammeled with conditions for two or three years, and, frequently, they are only saved by particular and special attention, an inherent strong desire to work, and unusual ability to grasp and imbibe instruction.

We recognize that much may be done in the direction of improving conditions, but feel that the Association of Dental Faculties deserves credit for having done more to elevate the dental profession and dental educational matters than any other body, and future improvement lies in the better understanding and obedience to rules as they exist, not in further obligations and multiplication of suggestions.

Let men like Dr. Patterson, whose earnest devotion to dental educational matters is fully appreciated, exert their influence in the direction of a better apprehension and a more thorough carrying out of the rules of the association, and much will be accomplished.—L. Greenbaum, in *Stomatologist.*

